# A novel *BRD4-NUT* fusion in an undifferentiated sinonasal tumor highlights alternative splicing as a contributing oncogenic factor in NUT midline carcinoma

**DOI:** 10.1038/oncsis.2015.33

**Published:** 2015-11-09

**Authors:** A Stirnweiss, K McCarthy, J Oommen, M L Crook, K Hardy, U R Kees, S D Wilton, A Anazodo, A H Beesley

**Affiliations:** 1 Division of Children's Leukaemia and Cancer Research, Telethon Kids Institute, The University of Western Australia, West Perth, WA, Australia; 2 Kids Cancer Centre, Sydney Children's Hospital, Randwick, NSW, Australia; 3 Department of Pathology, Princess Margaret Hospital for Children, Perth, WA, Australia; 4 Cyto Labs Pty Ltd, Perth, WA, Australia; 5 Molecular Therapy Laboratory, Western Australian Neuroscience Research Institute, Centre for Comparative Genomics, Murdoch University, Perth, WA, Australia; 6 Prince of Wales Hospital, Randwick, NSW, Australia

## Abstract

NUT midline carcinoma (NMC) is a fatal cancer that arises in various tissues along the upper midline of the body. The defining molecular feature of NMC is a chromosomal translocation that joins (in the majority of cases) the nuclear testis gene *NUT* (*NUTM1*) to the bromodomain protein family member 4 (*BRD4*) and thereby creating a fusion oncogene that disrupts cellular differentiation and drives the disease. In this study, we report the case of an adolescent NMC patient presenting with severe facial pain, proptosis and visual impairment due to a mass arising from the ethmoid sinus that invaded the right orbit and frontal lobe. Treatment involved radical resection, including exenteration of the affected eye with the view to consolidate treatment with radiation therapy; however, the patient experienced rapid tumor progression and passed away 79 days post resection. Molecular analysis of the tumor tissue identified a novel in-frame *BRD4-NUT* transcript, with *BRD4* exon 15 fused to the last 124 nucleotides of *NUT* exon 2 (*BRD4-NUT* ex15:ex2_Δnt1–585_). The partial deletion of *NUT* exon 2 was attributed to a mid-exonic genomic breakpoint and the subsequent activation of a cryptic splice site further downstream within the exon. Inhibition of the canonical 3′ acceptor splice site of *NUT* intron 1 in cell lines expressing the most common NMC fusion transcripts (PER-403, *BRD4-NUT* ex11:ex2; PER-624, *BRD4-NUT* ex15:ex2) induced alternative splicing from the same cryptic splice site as identified in the patient. Detection of low levels of an in-frame *BRD4-NUT* ex11:ex2_Δnt1–585_ transcript in PER-403 confirmed endogenous splicing from this alternative exon 2 splice site. Although further studies are necessary to assess the clinical relevance of the increasing number of variant fusions described in NMC, the findings presented in this case identify alternative splicing as a mechanism that contributes to this pathogenic complexity.

## Introduction

NUT midline carcinoma (NMC) is a particularly aggressive and fatal form of undifferentiated epithelial cancer affecting both children and adults.^1^ The genetic hallmark of this disease is a rearrangement of chromosome 15 - in the majority of cases fusing the testis-specific nuclear gene *NUT* (also known as *NUTM1* and *C15orf55*) to the bromodomain-containing gene *BRD4* (bromodomain protein family member 4) on chromosome 19 and thereby creating a new fusion protein that markedly disrupts squamous cell differentiation and promotes oncogenesis.^2, 3, 4^ Variant fusions between NUT and the bromodomain protein BRD3, or the nuclear receptor SET domain-containing protein NSD3 have also been described.^5, 6, 7^ Currently, little is known about the functionality of NUT beside its association with the histone acetyltransferase p300, which is thought to contribute to postmeiotic histone hyperacetylation and chromatin compaction in elongating spermatids.^2^ In contrast, BRD4 is an important member of the *b*romodomain and *e*xtra-*t*erminal domain proteins (the BET family) known to regulate cell cycle progression, survival signaling, chromatin structure, epigenetic memory and embryonic stem cell development.^8, 9, 10, 11^ This ubiquitously expressed transcriptional coactivator contains two bromodomains that enable BRD4 to recognize and bind epigenetic marks on DNA, and a domain at the C terminus that recruits the positive transcription elongation factor b (P-TEFb) and is thus critical for the assembly of the transcriptional machinery.^8, 10, 12^ Exactly how the fusion of those two proteins alters their biological function in the context of NMC however, is still not fully understood.

## Results and discussion

**Figure 1:**
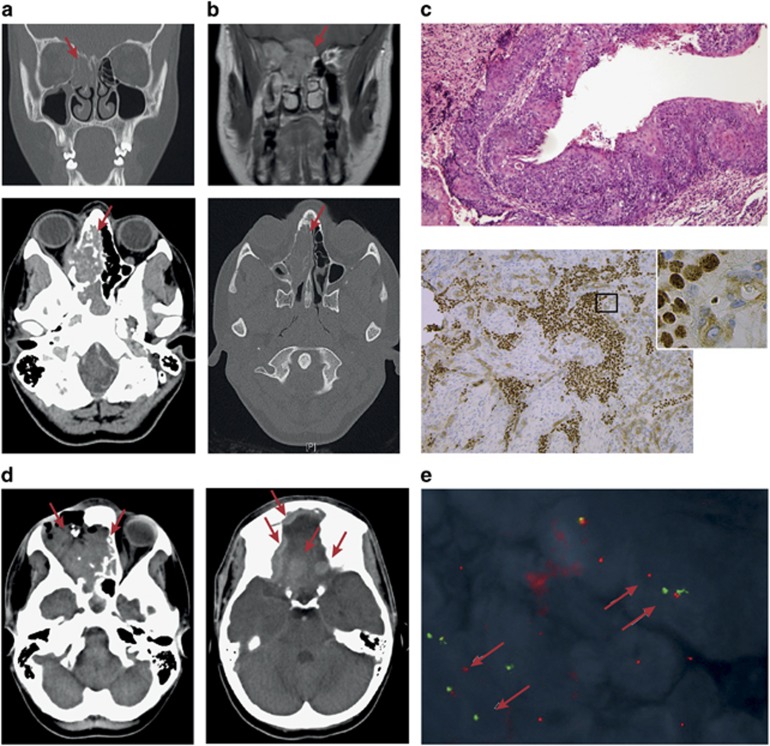
Case report of an adolescent diagnosed with NMC. (**a**) CT and (**b**) MRI imaging at presentation showed a mass in the superior nasal cavity, the right anterior ethmoid and the right anterior frontal lobe, further extending through the medial wall to the right orbit and into the right maxilla. (**c**) Histopathology of a diagnostic biopsy specimen. (Upper panel) Hematoxylin and eosin staining showed characteristics of undifferentiated carcinoma with nests of abrupt squamous differentiation. (Lower panel) Immunohistochemistry staining with a NUT-specific antibody (C52B1; Cell Signaling Technology, Boston, MA, USA). (**d**) CT images obtained 41 days after radical resection of the original tumor showed disease progression within the surgical site, nasal cavity and ethmoid sinus with extension into the anterior cranial fossa and into the left orbit impinging upon the left optic nerve. (**e**) Fluorescence *in situ* hybridization with BAC clones binding chromosome 15q14 upstream (RP11-74D7, RP11-88A04 and RP-11-242K3 labeled with SpectrumGreen Vysis; Abbott Molecular, Des Plaines, IL, USA) and downstream (RP-11-1H8 and RP11-477L8 labeled with Spectrum Orange Vysis; Abbott Molecular) of the *NUT* gene confirmed the diagnosis of NMC.

A 14-year-old girl presented to a local emergency department with severe right facial pain and visual disturbance. On examination there was altered sensation over the right cheek, diplopia, ptosis, proptosis and significant visual impairment of the right eye. Magnetic resonance imaging (MRI) and computed tomography (CT) scans demonstrated an irregular destructive mass involving the superior nasal cavity, the right anterior ethmoid and right maxilla, extending through the medial wall of the right orbit, compressing and invading the medial rectus with pressure effect and displacement of the optic nerve. Additionally, the cribiform plate was destroyed and there was involvement in the right frontal lobe (Figures 1a and b). There was no evidence of distant metastasis on positron emission tomography or abdominal and chest CT scans (data not shown). Detailed histological examination of a biopsy specimen identified an undifferentiated tumor with high nuclear:cytoplasmic ratio and a high mitotic rate, arranged in nests with abrupt squamous differentiation (Figure 1c, upper panel). Further immunohistochemistry provided no evidence for the presence of neuroendocrine differentiation markers (data not shown) but revealed positive staining for NUT (Figure 1c, lower panel). Fluorescence *in situ* hybridization showed a separation of probes targeted proximal and distal to the *NUT* gene (Figure 1e), confirming the diagnosis of NMC. The rapid diagnosis allowed enrollment on and consultation with the International NMC Registry at the Dana-Farber Cancer Institute, providing access to a current data set that was used to guide treatment. Forty-one days after the patient underwent radical resection including exenteration of the affected eye, she re-presented with significantly increased facial pain, swelling, clear rhinorrhea and impairment of vision in the left eye. CT imaging showed disease progression within the surgical site, nasal cavity and ethmoid sinus with extension into the anterior cranial fossa and into the left orbit, impinging upon the left optic nerve (Figure 1d). Access to experimental BET inhibitor treatment was unfortunately not possible owing to legislative, geographical and financial issues, as well as limitations of clinical trial design - problems that are not uncommon in the wider Australian adolescent and young adult cancer population.

**Figure 2:**
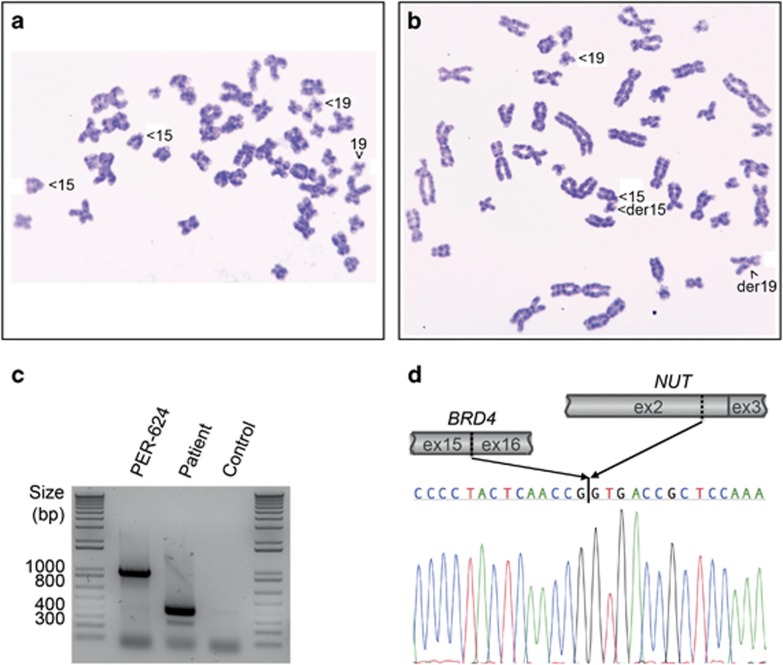
Identification of a novel *BRD4-NUT* fusion transcript. (**a**,**b**) Cytogenetic analysis of a biopsy specimen obtained at diagnosis. Images show representative metaphases of (**a**) normal and (**b**) tumor cells. (**c**) RT–PCR using primers targeted to *BRD4* exon 15, and *NUT* exon 3 (see Supplementary Table 2) identified an approximately 600 bp shorter *BRD4-NUT* product in the patient sample compared with that of the cell line PER-624, which is known to expresses a *BRD4-NUT* ex15:ex2 fusion transcript. Methods: Total RNA was extracted in TRIzol (Life Technologies, Carlsbad, CA, USA), purified using the RNeasy Mini Kit (Qiagen, Valencia, CA, USA) and reverse transcribed using the SuperScript VILO cDNA Synthesis Kit (Life Technologies). PCR products were amplified using GoTaq Flexi DNA polymerase (Promega, Madison, WI, USA) and purified with the QIAquick Gel Extraction Kit (Qiagen). (**d**) Sanger sequencing of the RT–PCR product amplified from patient-derived RNA identified a unique transcript with *BRD4* exon 15 fused to the last 124 nucleotides of *NUT* exon 2 (i.e., with deletion of the first 585 nucleotides of exon 2).

The patient passed away 98 days after her original biopsy. Cytogenetic analysis of viable cells from a post-mortem sample revealed a t(15;19)(q14;p13.13) rearrangement consistent with the presence of a *BRD4-NUT* fusion (Figure 2b). RNA analysis via reverse transcriptase PCR (RT–PCR) identified the transcript fusion position to be downstream from *BRD4* exon 15 and upstream of *NUT* exon 3. However, the RT–PCR product was 600 bp shorter than that amplified from an NMC cell line that expresses a *BRD4-NUT* ex15:ex2 fusion transcript (PER-624; Figure 2c). Subsequent Sanger sequencing of the RT–PCR product confirmed that the patient-derived tumor cells expressed a novel in-frame *BRD4-NUT* fusion transcript with the last 124 nucleotides (nt) of *NUT* exon 2 fused to *BRD4* exon 15 (*BRD4-NUT* ex15:ex2_Δnt1–585_; Figure 2d). This is the first report of an NMC case where the *BRD4-NUT* transcript does not contain the entire sequence of *NUT* exon 2. At the protein level, this leads to a disruption of the proline-rich domain NUT_N (amino acids (aa) 8–332) while the previously described p300-binding domain (aa 346–593) remains intact.^13^ The fusion protein should thus retain its ability to sequester the histone acetyltransferase p300 - a central activity of the fusion protein that results in foci of chromatin hyperacetylation and impaired transcription of genes driving differentiation.^4, 6, 14^ Indeed, immunohistochemistry of the tumor demonstrated the punctate nuclear staining of the fusion protein that is characteristic for NMC (Figure 1c, lower panel).

**Figure 3:**
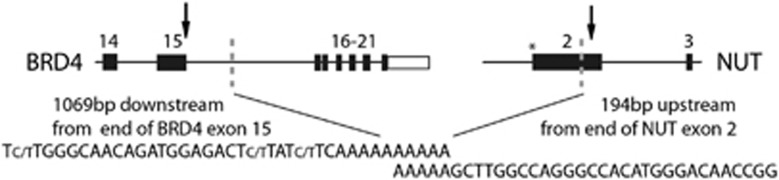
Schematic representation of the genomic rearrangements in the index case. Genomic breakpoints (dashed lines and indicated sequences) are located ~1 kb downstream from the end of *BRD4* exon 15 and 515 bp downstream from the start of *NUT* exon 2, resulting in the depletion of the canonical 3′ acceptor splice site of *NUT* intron 1 (asterisk). Arrows illustrate the position of the corresponding RNA breakpoints. Alternative nucleotides within the indicated DNA sequence (e.g., C/T) indicate heterogeneity at that position. Methods: Genomic breakpoints were amplified by nested PCR using LongAmp DNA polymerase (New England BioLabs Inc., Ipswich, MA, USA) and the primer pairs are described in Supplementary Table 2. The PCR product was purified using the QIAquick Gel Extraction Kit (Qiagen) and analyzed via Sanger sequencing.

The partial deletion of *NUT* exon 2 within the fusion transcript indicated the potential activation of a cryptic splice site within this exon. *In silico* analysis of *NUT* exon 2 and 100 bp of the flanking introns using the online tool ESEfinder 3.0 (see Cartegni *et al.*^15^) predicted 11 potential splice sites, including the canonical *NUT* intron 1 acceptor splice site, and an internal cryptic splice site that corresponds with the position of the fusion transcript breakpoint in the described NMC case (Table 1). To test whether the upstream canonical acceptor site may have been deleted as part of the translocation event, we used nested PCR primers to amplify the genomic region between *BRD4* exon 15 and *NUT* exon 3. Sanger sequencing of this product demonstrated the genomic breakpoint to also be within *NUT* exon 2 but 70 bp upstream from the RNA breakpoint (Figure 3). Hence, the genomic breakpoint within *NUT* is mid-exonic and results in the deletion of all predicted acceptor splice sites in *NUT* exon 2 except for the implicated cryptic site (Table 1). Even though use of this cryptic splice site has not been reported before, RNA splicing is known to have a key role in generating NMC fusion transcripts; at a chromosome level, *NUT* exon 1 is intact in all other fusion genes so far described and its removal via splicing is therefore essential to maintain an open reading frame.^3, 16^ Importantly, the use of the cryptic splice site within *NUT* exon 2 maintains this open reading frame, whereas complete removal of exon 2 (and thus direct fusion of *BRD4* to *NUT* exon 3) would result in premature truncation of the transcript.

**Figure 4:**
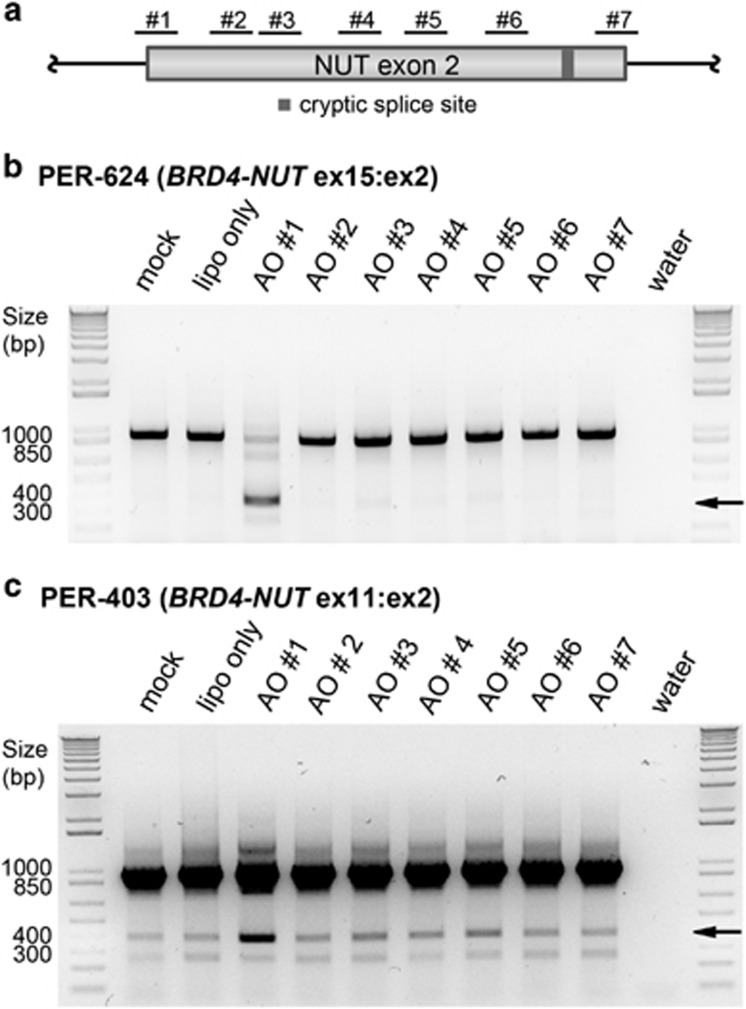
Functional evaluation of *in silico* predicted splice sites in *NUT* exon 2. (**a**) Illustration of AO binding sites, not drawn to scale. The AOs were synthesized as 2′-O-methyl modified bases on a phosphorothioate backbone^26^ and designed to target splice sites and enhancer elements of *NUT* exon 2; AO sequences are described in Supplementary Table 2. (**b**,**c**) Inactivation of the canonical acceptor splice site (AO no. 1; lane 3) leads to a deletion of the first 585 nucleotides of *NUT* exon 2 (confirmed by Sanger sequencing). Arrows indicate the size of the corresponding alternative splicing products. AOs were introduced into two NMC cell lines (**b**) PER-624 and (**c**) PER-403 (representative of different *BRD4-NUT* fusion variants) to manipulate normal splicing. Methods: Cells were seeded 24 h before their transfection with 100 nm AO using the Lipofectamine RNAiMAX Reagent (Life Technologies) according to the manufacturer's protocol. Untreated cells (mock; lane 1) and cells treated with Lipofectamine alone (lipo only; lane 2) were used as controls. RNA was extracted from each of the samples and converted into cDNA as described in Figure 3. The sequence between *BRD4* exon 15 and *NUT* exon 3 was amplified using the GoTaq Flexi DNA polymerase (Promega) system and the primers described in Supplementary Table 2.

To further examine the role of *NUT* exon 2 splicing in NMC, we generated antisense oligomers (AOs) to block predicted splice sites (Table 1) and exonic splicing enhancers (Supplementary Table 1). We analyzed the effects of seven different AOs (Figure 4a) in two cell lines that represent the most common *BRD4-NUT* variants in NMC (PER-403, ex11:ex2; PER-624, ex15:ex2).^3, 17^ In both cases, only AO no. 1, targeting the canonical *NUT* intron 1/exon 2 acceptor site, induced alternative splicing (Figures 4b and c). Sanger sequencing of the corresponding RT–PCR products from both cell lines identified transcripts missing the first 585 nt of *NUT* exon 2 (PER-403, *BRD4-NUT* ex11:ex2_Δnt1–585_; PER-624, *BRD4-NUT* ex15:ex2_Δnt1–585_), thus confirming the activation of the same cryptic 3′ acceptor splice site observed in the index case. Although different *BRD4* exons are involved, the resulting fusions in both cell lines remain in frame. We did not observe any changes in phenotype in either cell line correlated with this splicing switch, in terms of growth rate or differentiation status (Ki67 or cytokeratin staining), consistent with the clinical observation that the *NUT* ex2_Δnt1–585_ fusion variant remains highly oncogenic (i.e. the patient succumbed to disease 98 days after diagnosis). Furthermore, low endogenous levels of *BRD4-NUT* ex11:ex2_Δnt1–585_ fusion transcript were detected in PER-403 (Figure 4c, lipofectamine and mock control lanes; breakpoint confirmed by sequencing), indicating that use of the cryptic splice site in *NUT* exon 2 may occur naturally in other NMC cases. Over the past decade, it has become evident that alteration of normal splicing patterns in tumors can support the progression to a more aggressive phenotype. The functions promoted by cancer-specific isoforms range from antiapoptotic and proproliferative (e.g., *EGFR*, *BCL-X*_*s*_, *BRAF*), to angiogenic (*VEGF-A*), hyperenergetic (*PKM*, *LDHC*), immune modulative (*HLA-G*, *MHC-I*) and prometastatic (*TGF-β, CDH1*, *FGFR2*).^18^ The expression of multiple fusion isoforms within NMC could therefore have important implications for tumor phenotype as well as the design of targeted therapies.

For more than 20 years, the predominant oncogenic variant in NMC was thought to involve the fusion of *BRD4* exon 11 to the start of *NUT* exon 2. Only recently, two additional *BRD4-NUT* isoforms have been described (*BRD4-NUT* ex14:ex2 and ex15:ex2), which could potentially indicate the existence of clinically relevant NMC subtypes.^3, 19^ In this study of an adolescent NMC patient presenting with an undifferentiated sinonasal tumor, we identify a fourth *BRD4-NUT* fusion variant (*BRD4-NUT* ex15:ex2_Δnt1–585_). This is the first described case with a partial deletion of *NUT* exon 2, which disrupts a proline-rich protein domain but without ameliorating oncogenicity. NMC is an extremely aggressive disease that is refractory to conventional treatments, yet significant preclinical and clinical responses have been reported for BET inhibitors and histone deacetylase inhibitors, making them promising candidates for NMC therapy.^2, 13, 20, 21^ As a result of these findings, phase I clinical trials have been opened that investigate the efficacy of different BET inhibitors (GSK525762, TEN-010, OTX015) and a dual phosphoinositide 3 kinase/histone deacetylase inhibitor (CUDC-907) in NMC and other advanced cancers. It will be some time before the results of these trials are known; however, there is evidence that molecular factors such as the dependency of the tumor on *MYC* signaling^22, 23, 24^ are likely to affect the efficacy of BET inhibitors in different cancer settings. Indeed, we have previously reported that the cytotoxicity of the BET inhibitor JQ1 may differ substantially between NMC subtypes,^25^ although it should be noted that observation was limited to a small number of cell lines and did not examine the effects of this drug class on cellular differentiation. The relationship between the efficacy of BET inhibitors and the genetic features of NMC (e.g., fusion type and co-operating mutations), its molecular features (e.g., *MYC* expression), or its cell of origin, has not yet been described, hence there is a continued need for a better understanding of the cellular processes altered by NUT fusion proteins to develop optimal treatment strategies for NMC patients.

## Figures and Tables

**Table 1:** Potential splice sites within *NUT* exon 2 and 100 bp of the adjacent introns predicted by ESEfinder 3.0

*Nt*^a^	*Motif*	*Sequence (5′–3′)*	*Score*
−15	3′ acceptor splice site^b^	tctttgtctcaacagCATCTGCATTGCCGG	9.20
85	3′ acceptor splice site	CTTCTGACCCACCAGACCACCCACCCAGGG	8.92
142	3′ acceptor splice site	CAGTATTCTCTCCAGACAACCCTCTGATGC	7.74
173	3′ acceptor splice site	CTCTGCTTTCCCCAGCTCACTGTTGGTGAC	11.11
231	5′ donor splice site	GCTGGGGCTGGCAAGGTCATTGTCAAAGTC	5.79
260	5′ donor splice site	CAAGACAGAAGGGGGGTCAGCTGAGCCCTC	5.24
303	3′ acceptor splice site	TTTATCCTTACTCAGACTGCCCTCAATTCG	8.08
408	3′ acceptor splice site	ATTCTGCCCTCTAAGGCTGTTGGTGTCAGC	6.67
453	3′ acceptor splice site	GGCCTTCCGCCTCAGCCTCCACCACCAGTT	7.50
571	5′ donor splice site	CCAAGCCTTCCCTAGGTGACCGCTCCAAAA	6.21
571	3′ acceptor splice site^c^	CCAAGCCTTCCCTAGGTGACCGCTCCAAAA	6.98
695	5′ donor splice site^b^	TTCCTGTTTTCTTATGTAAGTGGGGAGACC	5.48

Abbreviation: Nt, nucleotide.

a Nt positions relative to the start of *NUT* exon 2, intronic sequences are in lowercase and exonic sequences in uppercase.

b Canonical splice site.

c Cryptic splice site that has been activated in the index case.
